# The persistence of pesticides in atmospheric particulate phase: An emerging air quality issue

**DOI:** 10.1038/srep33456

**Published:** 2016-09-15

**Authors:** Joanna Socorro, Amandine Durand, Brice Temime-Roussel, Sasho Gligorovski, Henri Wortham, Etienne Quivet

**Affiliations:** 1 Aix Marseille Univ, CNRS, LCE, Marseille, France

## Abstract

The persistent organic pollutants (POPs) due to their physicochemical properties can be widely spread all over the globe; as such they represent a serious threat to both humans and wildlife. According to Stockholm convention out of 24 officially recognized POPs, 16 are pesticides. The atmospheric life times of pesticides, up to now were estimated based on their gas-phase reactivity. It has been only speculated that sorption to aerosol particles may increase significantly the half‐lives of pesticides in the atmosphere. The results presented here challenge the current view of the half-lives of pesticides in the lower boundary layer of the atmosphere and their impact on air quality and human health. We demonstrate that semivolatile pesticides which are mostly adsorbed on atmospheric aerosol particles are very persistent with respect to the highly reactive hydroxyl radicals (OH) that is the self-cleaning agent of the atmosphere. The half-lives in particulate phase of difenoconazole, tetraconazole, fipronil, oxadiazon, deltamethrin, cyprodinil, permethrin, and pendimethalin are in order of several days and even higher than one month, implying that these pesticides can be transported over long distances, reaching the remote regions all over the world; hence these pesticides shall be further evaluated prior to be confirmed as POPs.

Aerosol particles are omnipresent in the lower boundary layer of the atmosphere and exert an important influence on the global climate and human health. Aerosol particles have typical atmospheric lifetimes of about 3 to 10 days. Considering the fact that air masses can be transported over several thousand kilometers in period of two weeks, there are really no places where we can expect to find truly remote conditions, especially in the Northern Hemisphere. Lelieveld *et al*.^1^ have shown that outdoor air pollution, mostly by particulate matter (PM) 2.5, leads to 3.3 million premature deaths per year worldwide, predominantly in Asia, a figure that could double by 2050 if emissions continue to rise at the current rate. Atmospheric aerosols may affect the human health especially the particles with diameter smaller than 100 nm exhibit more adverse health effects compared to the larger particles due to the higher probability to penetrate in the human lung and even in the blood^2^.

During applications, a significant fraction of applied pesticides, about 15 to 40%, is dispersed in the atmosphere by volatilization or spray drift processes^3^,^4^. Pesticides travel in the atmosphere with long range atmospheric transport and deposition from their emission area^5^,^6^. The fate of pesticides is influenced by their partition between the gas phase and particulate phase. Considering the low volatility of majority of the commonly used pesticides, they are often adsorbed on the surface of atmospheric particles^7^,^8^. They may undergo different transport and transformation processes resulting in the generation of secondary products that could be even more hazardous than the primary emitted pesticides.

These aspects are central to atmospheric composition changes, air quality and associated climate change. Hence, a better chemical characterization of the processes associated with the adsorbed pesticides on atmospheric aerosols is highly desirable to understand the variable chemical and physical controlling factors allowing assessment of the contributions and consequences of global environmental change.

According to Stockholm convention^9^ (a treaty to protect human health and the environment from POPs), the organic compounds with half-life time (t_½_) greater than 2 days in air are considered as persistent organic pollutants (POPs). The Stockholm convention considers the gas-phase reactions toward hydroxyl radicals (OH) as a major degradation pathway of pesticides in the atmosphere^10^. Therefore, the atmospheric half-lives of pesticides are calculated from the gas-phase reactivity with respect to the OH radicals, using structure–activity relationships (SAR) used by U.S. Environmental Protection Agency, software AOPWIN (Atmospheric Oxidation Program)^11^.

However, the heterogeneous reactions of pesticides which occur on the surface of atmospheric aerosols may proceed at different rates than the gas-phase reactions. Indeed, Socorro *et al*.^12^ have shown that half-lives of 8 commonly used pesticides span from 9 to >24 days for an atmospheric ozone level of 9.8 · 10^11^ cm^−3^, demonstrating that these species are very persistent regarding the ozone (O_3_) reactivity on atmospheric particulate phase^13^. In this context, it is essential to investigate the fate of pesticides adsorbed on aerosol particles to determine their persistence in the atmosphere with respect to the highly reactive hydroxyl radicals. Indeed, OH radicals are considered as the dominant oxidizing and cleansing agent determining the oxidative capacity of the atmosphere.

In the present study we investigated the heterogeneous reactivity of eight pesticides, difenoconazole, tetraconazole, fipronil, oxadiazon, deltamethrin, cyprodinil, permethrin, and pendimethalin enriched in atmospheric particulate phase toward gas phase OH radicals. The emerged results strongly indicate that these commonly used pesticides once adsorbed on the atmospheric aerosols can be transported thousands of kilometres far away from the place where have been applied.

From the environmental pollution point of view these results are extremely important and it should be considered while developing appropriate environmental strategies which in turn will contribute to better describe and understand the atmospheric behavior of pesticides and their persistence in the environment.

## Results and Discussion

Silica is commonly a major constituent of mineral dust^14^ on which surface the organic compounds are adsorbed and (photo)oxidized during their transport^15^. To study the OH heterogeneous kinetic reactions the pesticides were coated on silica particles (AEROSIL R812). Silica particles are characterised by a high surface to volume ratio which makes them an ideal proxy for investigation of heterogeneous reactions. The pesticides adsorbed on silica particles were simultaneously oxidized by gas-phase OH radicals and gas-phase O_3_ which is necessary for the production of OH radicals as explained in the supplementary information.

The pseudo-first-order rate constants for the heterogeneous oxidation of the particle-phase pesticides induced by OH radicals (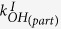) were determined by analysing the temporal profiles from 0 to 6 h obtained for six different OH radicals concentrations (3 · 10^7^; 6.1 · 10^7^; 8 · 10^7^; 9.3 · 10^7^; 1.4 · 10^8^ and 1.5 · 10^8^ cm^−3^).

**Figure 1:**
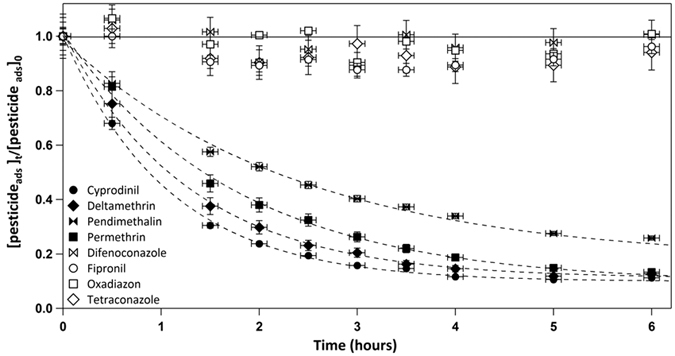
Temporal profile of the normalized concentrations of eight studied pesticides to both OH and O_3_ reactivity for an OH concentration of 1.5 · 10^8^ cm^−3^ and 1.7 · 10^14^ cm^−3^ of ozone for a period of 6 hours. Dotted curves represent the exponential fit of the experimental points. The horizontal error bars corresponds to the sampling time of 10 min of particles during the experiment and the error bars of the pesticide concentrations are the standard deviations calculated for three injections of the sample t = 0 h in GC-(QqQ)-MS/MS.

Figure 1 shows, as an example, the exponential decays of the pesticides applying the highest OH and O_3_ concentrations, 1.5 · 10^8^ cm^−3^ and 1.7 · 10^14^ cm^−3^, respectively.

One experimental limitation in the laboratory kinetic experiments is to reproduce realistic OH concentrations in order of 10^6^–10^7^ cm^−3^. The first two applied OH concentrations, i.e., 3 · 10^7^ and 6.1 · 10^7^ cm^−3^, correspond to the highly polluted regions^16^. Although the other applied OH concentrations, i.e., 8 · 10^7^; 9.3 · 10^7^; 1.4 · 10^8^ and 1.5 · 10^8^ cm^−3^, were 1–2 orders of magnitude higher than the global average OH concentrations^16^, four of the considered pesticides (difenoconazole, tetraconazole, fipronil, and oxadiazon) were almost not oxidized at all (Fig. 1). The other four pesticides (deltamethrin, cyprodinil, permethrin, and pendimethalin) were slightly degraded in the following manner from the fastest to the slowest: cyprodinil > deltamethrin > permethrin > pendimethalin.

A detailed procedure describing the calculations of the second order rate constants 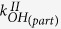 for the heterogeneous reactions of OH radical with cyprodinil, deltamethrin, permethrin, and pendimethalin is given in the SI Appendix.

**Figure 2:**
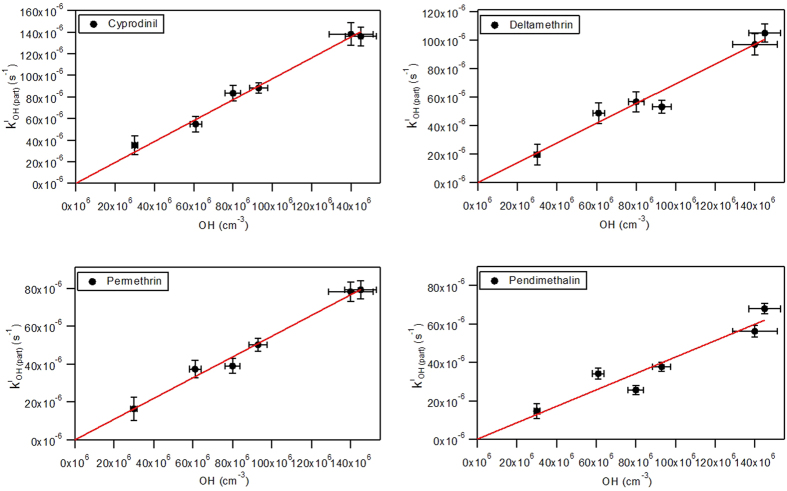
The observed pseudo-first order rate constants 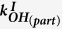 in function of OH radical concentrations. The errors for *k*^*I*^_*OH (part)*_ were obtained from the statistical errors of the linear fit. The uncertainties are standard deviations estimated by the Igor Pro software (version 6.3.5.5). The relative OH radical concentration uncertainties were obtained by the sum of the relative uncertainties of mean m-xylene concentrations and rate constant of the reaction between m-xylene and OH radicals (see Eq. S8).

In order to determine the second order rate constant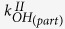, the experimental pseudo first order reaction rate constants 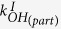 were plotted as a function of the OH radical concentrations (Fig. 2).

For the heterogeneous reactions of O_3_ with of cyprodinil, deltamethrin, permethrin, and pendimethalin, the following second order rate constants (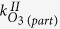), (8.8 ± 0.2) · 10^−19^, (8.0 ± 0.2) · 10^−19^, (6.0 ± 0.2) · 10^−19^ and (3.4 ± 0.1)·10^−19^ cm^3^ molecule^−1^ s^−1^ 12, were used in Eq. S12. The intercept of the plot depicted in Fig. 2 corresponds to (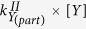) which was then subtracted, following the Eq. S12, to finally obtain the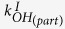.

The linearity of the kinetic data depicted in Fig. 2 corresponds to a Langmuir-Rideal mechanism^17^,^18^,^19^. In Fig. 2 we assume that the linearity will continue down to ambient OH concentrations. Considering the extremely short life time of OH radicals comprised between 0.01 s and 1 s^16^, it is logical to expect direct bimolecular collision between the gas-phase OH radicals and the particulate pesticides without the adsorption step of OH radical on the surface of particles which would correspond to Langmuir-Hinshelwood mechanism^12^.

These results are in contrast with recent studies^20^,^21^,^22^,^23^ related to the heterogeneous OH reactions with liquid surfaces which proceed through Langmuir-Hinshelwood mechanism. The different behavior of the kinetic data could be ascribed to the different surfaces i.e. solid versus liquid film.

A study on the dependence of the first order rate coefficients of the deposited sample mass on the particles surface would be important to distinguish between the both mechanisms. Also, the experimental set-up is unable to generate low OH concentrations; hence, the parameter space of OH concentrations is not sufficient. However, even if the slope changes in Fig. 2 and kinetics behave more like Langmuir-Hinshelwood, the rates at ambient OH concentrations will be lower implying even longer half-lives of the pesticides.

The obtained 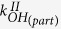 for the four pesticides span in the same order of magnitude, i.e., (4.3 ± 1.1)·10 ^−13^ and (9.7 ± 0.7)·10^−13^ cm^3^ molecules^−1^ s^−1^ (Table 1).

According to the Stockholm convention^9^, the classification of POPs is made by use of AOPWIN software. Basically, the program calculates the first order rate constants (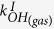

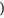
 by multiplying the second order rate constants (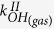) for the reaction of OH radicals with the organic compounds in the gas phase by an average OH radical concentration of 1.5 · 10^6^ cm^−3^ and for an exposure of 12 h by day which corresponds to 7.5 · 10^5^ cm^−3^ day^−1^ 24,25. It follows from Eq. 1 the half-life (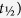:


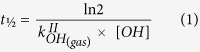


However, most of the pesticides represent the compounds with low vapor pressure (below 1 Pa) and thus a significant fraction of pesticides is enriched into atmospheric particulate phase. It was already speculated by Scheringer^26^ that the adsorption of semivolatile compounds on the atmospheric particles may significantly increase their half‐lives. This implies that in reality the half‐lives of the pesticides can be much longer than the current estimates by AOPWIN. In addition, the estimations made by AOPWIN gave unrealistically high rate constants for complex and highly chlorinated chemicals^27^,^28^.

Since the oxidation by OH radicals was considered as the most effective removal pathway of the pesticides, Scheringer *et al*.^29^ suggested a model to describe the reactivity of pesticides with respect to the OH radicals in both the gas phase and the particulate phase (Eq. 2).


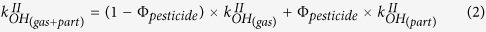


where 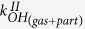 is the effective OH rate constant, Φ_pesticide_ represents the fraction of the adsorbed pesticides, 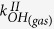 is the rate constant for the reaction between the OH radical and the pesticides dispersed in the gas phase, 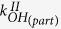 is the rate constant describing the reactions between OH radical and the pesticides adsorbed on the surface of atmospheric particles.

The fraction of pesticides adsorbed on the surface of particles, Φ_pesticide_, can be determined by AEROWIN software based on the Junge-Pankow partitioning model^30^:


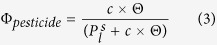


where c is a factor that depends on the excess heat of desorption from the particle surface (17.2 Pa cm), Θ is the surface of particles per unit of air volume (6.3 · 10^−6^ cm^2^ m^−3^) and 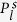 (Pa) is the vapor pressure of considered pesticide at 25 °C.

The estimated Φ_pesticide_ values, the effective rate constants and the calculated half-lives of the eight studied pesticides with respect to the OH reactivity are summarized in Table 1.

The partitioning between the gas phase and particulate phase is largely variable from 0.01 for pendimethalin up to 0.99 for difenoconazole. Therefore, Φ_pesticide_ should be taken into account while estimating the half-life of the pesticides.

Table 1 shows that pendimethalin and cyprodinil are predominantly found in the gas phase; thus the gas phase reactivity towards OH radicals contributes essentially to the half-lives of those pesticides.

Tetraconazole and oxadiazon partition more or less equally between the gas phase and the particulate phase. Considering the OH reactivity in both phases, these pesticides exhibit half-lives much higher than 1 day which implies long-range transport.

On the other hand, the estimated Φ_pesticide_ values of deltamethrin and permethrin indicate that these two pesticides are mostly enriched in the particulate phase. The experimentally measured rate constants 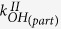 of these two pesticides are very close to the 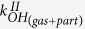 suggesting that the atmospheric half-lives are determined by the OH reactivity in the particulate phase. The half-lives of deltamethrin and permethrin are higher than 2 days strongly indicating that these two pesticides can be potentially considered as POPs according to the Stockholm convention.

The example of difenoconazole that is completely enriched in the particulate phase is especially striking with half-life much higher than 16 days with respect to the OH reactivity. Such long lived pesticides will be transported far from their place of release impacting the regional and global air quality, human health and wildlife.

**Figure 3:**
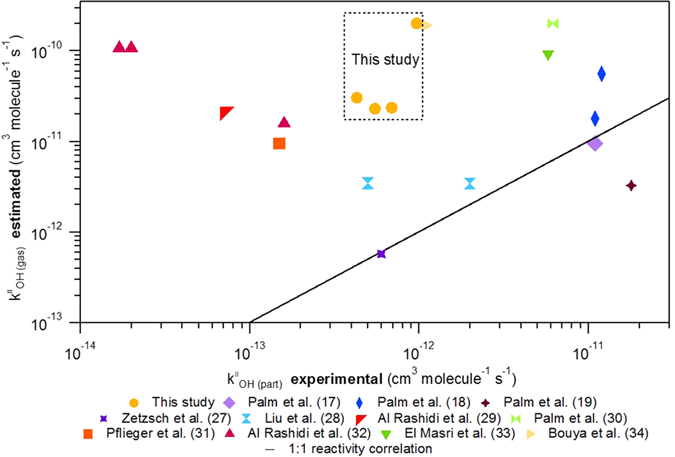
Rate constants estimated by “Atmospheric Oxidation Program (AOPWIN)” of gas phase OH radical reactivity in function of experimental rate constants of heterogeneous OH radical reactivity for number of pesticides.

Figure 3 graphically shows the difference between the OH reactivity towards pesticides dispersed in the gas phase and pesticides adsorbed on the atmospheric particles.

Almost all the experimental rate constants are clearly above the 1:1 correlation^17^,^18^,^19^,^31^,^32^,^33^,^34^,^35^,^36^,^37^,^38^ demonstrating that the experimental rate constants in the particulate phase are orders of magnitude slower than the estimated rate constants in the gas phase.

## Concluding remarks

Heterogeneous reactivity of OH radical with 8 commonly used pesticides was investigated.

In the past, it has been assumed that these pesticides are predominantly degraded by the OH radical in the gas phase. However, the majority of these pesticides are adsorbed on the atmospheric particle surface; thus their reactivity in particulate phase should be considered prior to take any conclusion about their half-lives and possible hazards.

Here, we confirm that OH rate constants for difenoconazole, tetraconazole, fipronil, oxadiazon, deltamethrin, cyprodinil, permethrin, and pendimethalin are few orders of magnitude lower than the estimated rate constants in the gas phase. This implies that the heterogeneous OH oxidation of pesticides adsorbed on atmospheric particles is a very slow process suggesting that the pesticides can persist long time in the atmosphere prior to be removed and transferred to terrestrial and aquatic ecosystems.

Therefore, the estimated half-lives of these pesticides based on AOPWIN estimations are not valid and should not be used during the preparation of adequate legislation.

The emerged kinetic data from this study can be of great help for further validation of AOPWIN program with more complex organic molecules containing more functional groups in order to increase the confidence in the accuracy of the half-lives estimations for pesticides.

## Materials and Methods

### Chemicals

Cyprodinil (purity 99.8%), deltamethrin (99.7%), difenoconazole (97.0%), fipronil (97.5%), oxadiazon (99.9%), pendimethalin (98.8%), permethrin (98.3%), and tetraconazole (99.0%) (PESTANAL^®^, analytical standard), 2,3-dimethyl-2-butene (99%), m-xylene (>99.5%) were purchased from Sigma-Aldrich and were used as received (Table S1).

### Silica particles coating

Commercial hydrophobic silica particles (AEROSIL R812, Degussa, purity SiO_2_ content ≥99.8%, average primary particle size of 7 nm and specific surface area (BET) of 260 ± 30 m^2^ g^−1^) were used as proxy of atmospheric mineral aerosols. Silica particles were coated with the pesticides according to a liquid/solid adsorption. 5 mL of pesticides solution at concentration 20 mg L^−1^ in dichloromethane (for HPLC, ≥99.8%, Sigma-Aldrich) was mixed with 500 mg of silica particles in a Pyrex bulb wrapped with aluminum foil. This bulb was ultrasonicated for 15 min. Then, dichloromethane was evaporated by a rotary evaporator (Rotavapor R-114, Büchi) at 40 °C and 850 ± 85 mbar. The load of pesticides on silica particles was about 0.02% by weight and the percentage of the coated aerosol surface was between 0.2 and 0.4%, less than a monolayer, assuming a uniform particle surface coverage as was detailed by Socorro *et al*.^12^.

### Production and measurements of OH radical

A HS-PTR-MS (High Sensitivity – Proton Transfer Reaction – Mass Spectrometer, Ionicon Analytik) was used to follow the concentrations of m-xylene and 2,3-dimethyl-2-butene (DMB). DMB is an alkene which produces OH radicals with a yield near unity through the reaction with ozone. m-xylene was used as a tracer to determine the OH radical concentrations.

The HS-PTR-MS allows on-line and continuous monitoring of organic compounds with detection limit of only few part par trillion (ppt). The ionization process is a soft process, meaning the energy transferred during the ionization is small (as compared to electron impact ionization) which limits the fragmentation of the initial compounds.

### OH radical reactivity

The coated powders of inert AEROSIL particles with pesticides were exposed in a rotating quartz bulb to six different OH radical concentrations (3 · 10^7^; 6.1 · 10^7^; 8 · 10^7^; 9.3 · 10^7^; 1.4 · 10^8^ and 1.5 · 10^8^ cm^−3^). This simplified method is a useful means to expose the pesticides at sub-monolayer thickness to reactive species from the gas phase surrounding the particles (such as ozone and OH radicals).

Detailed descriptions of materials and methods, silica particles coating, description of OH radical production and kinetic experiments, extraction and pesticide quantification, measurements of OH radicals and other procedures used are given in SI Appendix.

## Additional Information

**How to cite this article**: Socorro, J. *et al*. The persistence of pesticides in atmospheric particulate phase: An emerging air quality issue. *Sci. Rep.* **6**, 33456; doi: 10.1038/srep33456 (2016).

## Supplementary Material

Supplementary Information

## Figures and Tables

**Table 1:** Comparison of pesticides reactivity towards OH radicals in gas phase and in partficle phase (rate constants and half-lives) and partitioning of pesticides in particle phase with the effective rate constants.

Pesticide	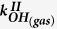 estimated in gas-phase^a^ (cm^3^ molecule^−1^ s^−1^)	Estimated half-lives in gas phase^b^ (days)	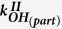 in particle phases (cm^3^ molecule^−1^ s^−1^)	Partitioning in particle phase^c^Φ_pesticide_	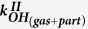 in gas and particle phases (cm^3^ molecule^−1^ s^−1^)	Atmospheric half-lives in gas and particle phases^b^ (days)
Pendimethalin	3.0 · 10^−11^	0.4	(4.3 ± 2.2) · 10^−13^	0.01	3.0 · 10^−11^	0.4
Cyprodinil	2.0 · 10^−10^	0.1	(9.7 ± 2.9) · 10^−13^	0.07	1.9 · 10^−10^	0.1
Tetraconazole	1.1 · 10^−11^	1.0	<<(4.3 ± 2.2) · 10^−13^	0.38	<<7.0 · 10^−12^	>>1.5
Oxadiazon	2.4 · 10^−11^	0.5	<<(4.3 ± 2.2) · 10^−13^	0.62	<<9.4 · 10^−12^	>>1.1
Fipronil	9.6 · 10^−11^	0.1	<<(4.3 ± 2.2) · 10^−13^	0.84	<<1.6 · 10^−11^	>>0.7
Deltamethrin	2.3 · 10^−11^	0.5	(6.9 ± 2.8) · 10^−13^	0.91	2.7 · 10^−12^	4.0
Permethrin	2.3 · 10^−11^	0.5	(5.5 ± 2.2) · 10^−13^	0.97	1.2 · 10^−12^	8.9
Difenoconazole	2.2 · 10^−11^	0.5	<<(4.3 ± 2.2) · 10^−13^	0.99	<<6.5 · 10^−13^	>>16.5

^a^Estimated rate constants by modeling using AOPWIN program.

^b^Half-lives for an average concentration [OH_(g)_] = 1.5 · 10^6^ cm^−3^ and for an exposure of 12 h by day.

^c^Partitioning in the particle phase estimated by AEROWIN software using the Junge-Pankow adsorption mode.
