# Supplementary material for: The persistence of pesticides in atmospheric particulate phase: An emerging air quality issue
Source: Sci Rep. 2016 Sep 15;6:33456. doi: 10.1038/srep33456 (PMC5024296; doi:10.1038/srep33456)
Supplement: Supplementary Information [file srep33456-s1.pdf]

The persistence of pesticides in atmospheric particulate phase: An emerging air quality issue

SUPPLEMENTARY INFORMATION

Joanna Socorro, Amandine Durand, Brice Temime-Roussel, Sasho Gligorovski, Henri Wortham, Etienne Quivet*

Aix Marseille Univ, CNRS, LCE, Marseille, France

*Corresponding author:

etienne.quivet@univ-amu.fr

Tel: + 33 4 13 55 10 54

__Chemical structures of the eight pesticides under study__

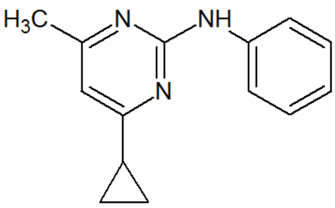

Cyprodinil

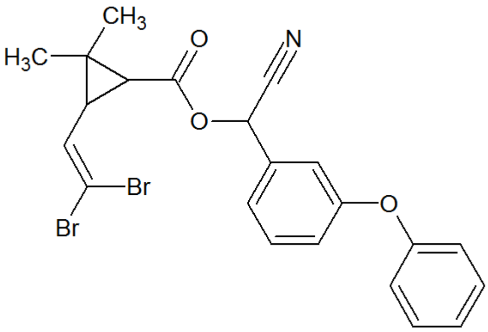

Deltamethrin

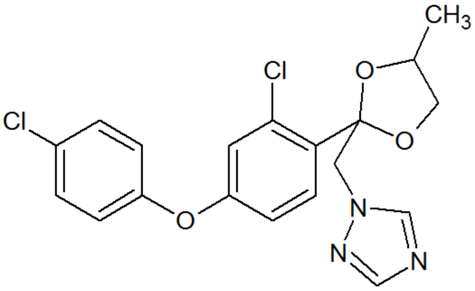

Difenoconazole

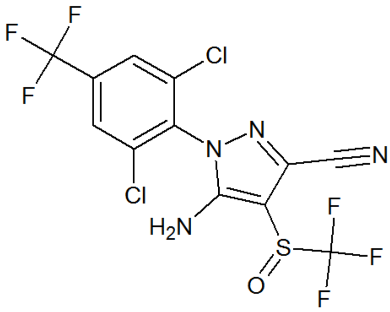

Fipronil

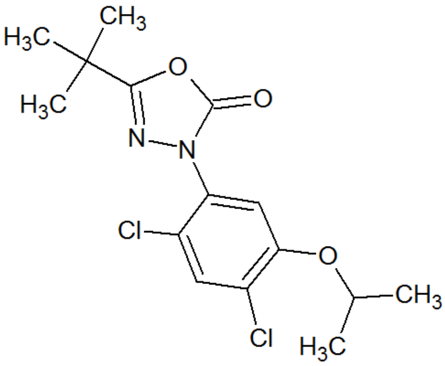

Oxadiazon

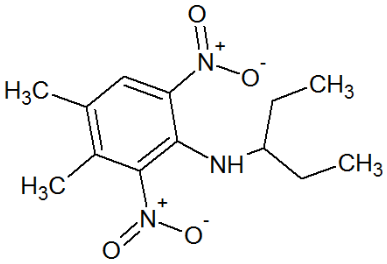

Pendimethalin

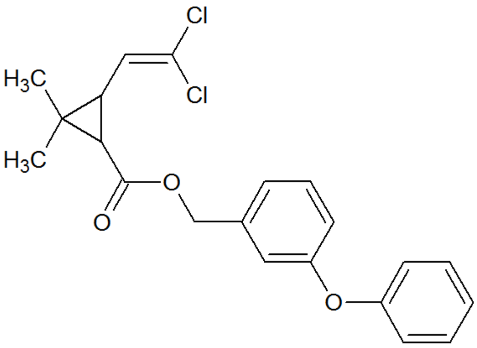

Permethrin

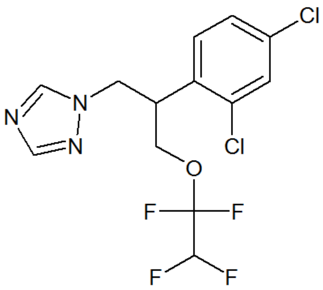

Tetraconazole

Description of the experiments: OH radical production and kinetic measurements

500 mg of dried silica particles coated by pesticides were placed inside a Pyrex bulb with the volume of 500 cm^3. The bulb was wrapped with aluminum foil to avoid any photo-degradation and was placed in a thermostated water bath at constant temperature (25 ± 1) °C under atmospheric pressure. The bulb was attached to a rotary evaporator (Laborota 4000 efficient, Heidolph) which ensured a homogeneous exposure of the particles during the experiment by rotation (Fig. S1).

Due to the yield near unity the ozonolysis of 2,3-dimethyl-2-butene (DMB) (35, 36) was chosen as a method to generate OH radicals into the bulb. This method allowed a stable production of OH radicals concentrations and easily maintainable during the experiments. The ozonolysis of 2,3-dimethyl-2-butene (Eq. S1) under dark conditions was previously developed by Lambe et al.[39]; and used by Le Person et al.[40] and Pflieger et al.[35].

O_3 + DMB → OH + Products $\qquad k_1 = (1.13 \pm 0.01)\cdot 10^{-15}$ cm^3 $molecule^{-1}$ s^{-1} [S1]

where k_1 is the average rate constant for the ozonolysis of DMB calculated from Jaspar et al.[41], Greene et Atkinson[42], and Witter et al.[43].

DMB reacts with OH radicals[44] as follows:

OH + DMB → Products $\qquad k_2 = (1.0 \pm 0.1)\cdot 10^{-10}$ cm^3 $molecule^{-1}$ s^{-1} [S2]

where k_2 is the average rate constant of the reaction calculated from Atkinson and Aschmann[45, 46], Atkinson et al.[47-49], and Otha and Ohyama[50].

Due to the extremely short lifetime of OH radicals, the reaction between the ozone and DMB occur directly inside the bulb. A constant DMB gaseous flow was generated from a permeation cell placed in a gas-phase generator (PUL 200, Saint Chamas, France) (Fig. S1). The permeation cell consisted of a permeable Teflon tube (perfluoroalkoxy, 6.4 mm i.d., 8 cm long) filled with a pure organic liquid DMB (2,3-dimethyl-2-butene (99 %) Sigma-Aldrich) and firmly closed from both sides. DMB was held in liquid/vapor phase equilibrium at 30 °C inside the

permeation cell. The permeation cell was placed in the permeation chamber (Fig. S1), which consisted of a stainless oven equipped with an inlet and outlet allowing a carrier gas flow (dried N_2) to pass through. N_2 was generated by a Domnick Hunter Nitrox UHPLCMS12 nitrogen generator, at 99.5 % purity (0.5 % O_2). A pure N_2 constant flow continuously passed at rate 54 mL min^{-1} through the Teflon porous cell to maintain a constant generation.

Ozone was generated with a constant flow of 65 mL min^{-1} by passing a flow of pure oxygen through an ozone generator (UVP, LLC Upland, UK). Oxygen flow was exposed to ultraviolet (UV) radiation at 185 nm by a mercury vapour pen ray lamp.

Each experiment was performed at constant ozone concentration of $1.7 \cdot 10^{14}$ molecule cm^{-3}. The ozone concentrations were monitored continuously and on-line by a photometric ozone analyser (O_3 41M, Environnement S.A). Since the ozone analyzer required a flow of 1300 mL min^{-1}, an additional flow of 850 mL min^{-1} was necessary because the maximum allowed gas flow in the reaction chamber is 500 mL min^{-1}. The both gaseous flows (DMB and ozone) were simultaneously inserted in the bulb reactor.

Simultaneously, a gaseous tracer, m-xylene was used to determine the OH radical concentrations (Eq. S3 and S4).

$$\text{OH} + \text{m-xylene} \rightarrow \text{Products} \qquad k_3 = (2.2 \pm 0.2) \cdot 10^{-11}\ cm^3\ molecule^{-1}\ s^{-1} \qquad [S3]$$

where k_3 is the average rate constant for the reaction calculated from Atkinson et al.[48], Otha and Ohyama[50], Hanse et al.[51], Doyle et al.[52], Lloyd et al.[53], Perry et al.[54], Davis[55], Cox and Derwent[56], Nicovitch et al.[57], Atkinson and Aschmann[58], Edney et al.[59], and Kramp and Paulson[60].

$$O_3 + \text{m-xylene} \rightarrow \text{Products} \qquad k_4 = 4 \cdot 10^{-21}\ cm^3\ molecule^{-1}\ s^{-1} \qquad [S4]$$

where k_4 is the average rate constant for the reaction calculated from Atkinson et al.[47] and Kramp and Paulson[60].

m-xylene was generated in the same manner as DMB using a permeation chamber at 50 °C supplied by a constant N_2 flow at 140 mL min^{-1}. Both DMB and m-xylene flows were diluted by a controlled N_2 flow prior to enter in the reaction chamber. The residence time of the gaseous species in the bulb reactor is 1 min.

A separate humidified air flow at rate of 270 mL min^{-1} was used to avoid a loss of ozone due its solubility in water. The relative humidity (RH) was adjusted by carrier gas N_2, which was split in two fluxes. The first one consisted of dry N_2 gas, while the second one was humidified by bubbling in deionized water. A mixing of these two flows at different ratios generated a carrier gas at controlled RHs. The relative humidity was kept constant at (55 ± 5) % for all the experiments. RH was measured throughout all the experiments by hygrometer “Hygrolog NT2” (Rotronic) with a “HygroClip SC04” probe (1.5 RH accuracy).

The silica particles coated with pesticides were exposed during a maximum period of 6 hours and 40 mg aliquot of particles were collected at regular intervals during this exposure.

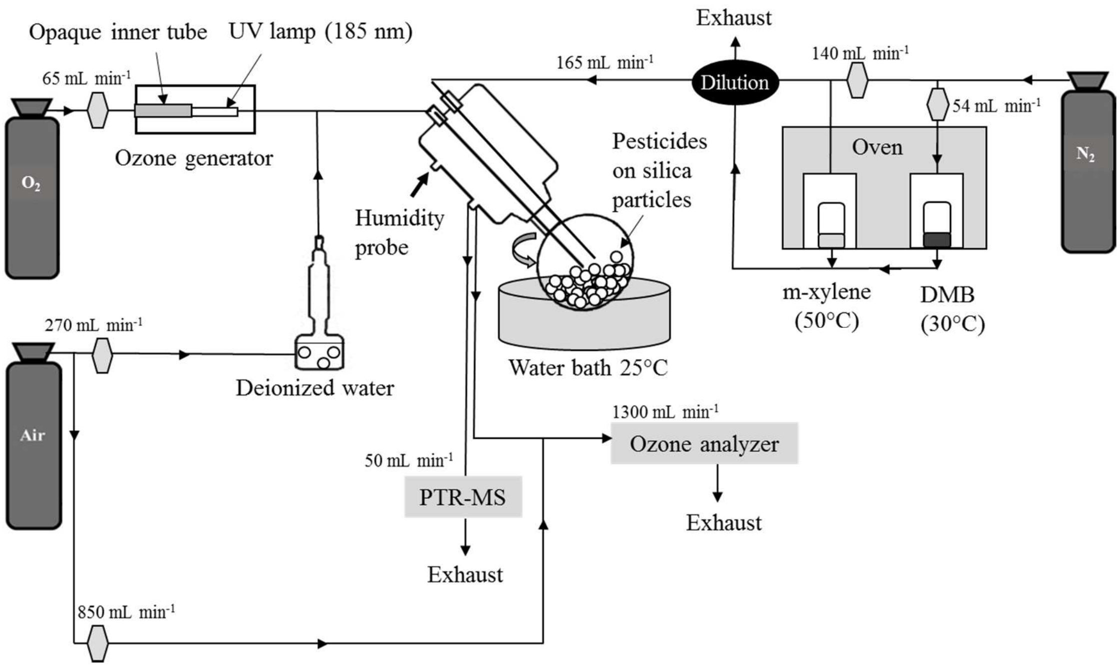

Figure S1: Experimental set-up to generate and monitor OH radicals and to evaluate the heterogeneous oxidation by OH radicals

Extraction and pesticide quantification

Following the exposure by OH radicals, pesticides adsorbed on silica particles were extracted by accelerated solvent extraction (ASE 300, Dionex). Each sample (40 mg of particles collected) was introduced in a 33 mL stainless-steel cell with an internal standard solution (Triphenyl phosphate, 99.9 %, Sigma-Aldrich). The optimized extraction conditions were as follows: extraction solvent, dichloromethane; oven temperature, 100 °C; pressure, 100 bars; heat up time, 5 min; static time 6 min. The flush volume amounted to 70 % of the extraction cell volume. The extracted pesticides, dissolved in dichloromethane, were purged from the sample cell using pressurized nitrogen (100 bars) for 300 s. Four cycles per cell were done.

Afterwards, the extracts were concentrated under a nitrogen flow using a concentration workstation (TurboVap II, Biotage) with pressure 11 bars and a water bath at 40 °C, until a 500 µL extract was obtained.

The obtained solutions were analysed by gas chromatography coupled to tandem mass spectrometry (GC-(QqQ)-MS/MS), with a Trace GC Ultra (Thermo Scientific) coupled to a TSQ Quantum™ Triple Quadrupole (Thermo Scientific) using electron impact ionisation (70 eV) according to the following parameters: column THERMO TG-5MS (internal diameter 0.25 mm, length 30 m, film thickness 0.25 µm), carrier gas: helium with 1 mL min^{-1} flow rate, split/splitless injector: splitless time of 2 min with surge pressure of 300 kPa during 2 min, injection volume: 1 µL, inlet temperature: 250 °C, interface temperature: 330 °C, with the following temperature program: hold 3 min at 75 °C; increase temperature to 180 °C at a rate of 25 °C min^{-1}; increase temperature to 300 °C at 5 °C min^{-1}; hold 3 min at 300 °C.

Monitoring of DMB and m-xylene and measurements of OH radicals

A HS-PTR-MS (High Sensitivity – Proton Transfer Reaction – Mass Spectrometer, Ionicon Analytik) was used to follow continuously DMB and m-xylene concentrations in gaseous phase

inside the bulb reactor with a time resolution of 15 s. The ionization process is a soft process, meaning the energy transferred during the ionization is small (as compared to electron impact ionization) which limits the fragmentation of the initial compounds. It consists of three parts: an ion source of H_3O^+, a reaction tube also called drift tube where H_3O^+ and followed compounds are contacted and a quadrupole analyzer. Ionization occurs by the chemical reaction of H_3O^+ ions with gas phase species having higher proton affinities than water. The proton transfer reaction takes place, as follows:

$$H_3O^+ + R \rightarrow RH^+ + H_2O \qquad [S5]$$

Parameters of the HS-PTR-MS during the experiments were as follow: E/N = 144 Td, U_{drift} = 600 V, T_{drift} = 60 °C, P_{drift} = 2.08 mbar. U_{drift} is the electric potential applied to the drift tube; T_{drift} and P_{drift} are the temperature and the pressure in the drift tube, respectively. E is the strength of the electrical field in V cm^{-1} and N is the gas number density in cm^3. The ratio E/N in Townsend (1 Townsend = 10^{-17} cm^2 V^{-1}) is a defining characteristic of the drift tube.

The OH radicals concentrations were determined using the consumption of the tracer m-xylene as follows.

$$-\frac{d[m-xylene]}{dt} = k_3 \times [m-xylene] \times [OH] + k_4 \times [m-xylene] \times [O_3] \qquad [S6]$$

Where k_3 and k_4 are the rate constants (Eq. S3 and S4)

The rate of the reaction between m-xylene and ozone (Eq. S4) is negligible compared to the rate of reaction between m-xylene and OH radicals (Eq. S3), and then Eq. S6 can be simplified in Eq. S7:

$$-\frac{d[m-xylene]}{dt} = k_3 \times [m-xylene] \times [OH] \qquad [S7]$$

The integration of the Equation S7 is done following Equation S8:

$$\int_0^t [OH] \times dt = \ln\left(\frac{[m-xylene]_0}{[m-xylene]_t}\right) \times \frac{1}{k_3} \qquad [S8]$$

Where t is the residence time of compounds in gaseous phase in the bulb, $[\text{m-xylene}]_0$ and $[\text{m-xylene}]_t$ are the initial and final concentrations of m-xylene in the bulb reactor and k_3 is the rate constant (Eq. S3)

Finally the Equation S8 allowed determining OH radical concentrations in the bulb reactor during the time experiment.

Heterogeneous reaction kinetics

To obtain the second order rate constants $k^{II}_{OH_{(part)}}$ for the heterogeneous reactions of OH radical with cyprodinil, deltamethrin, permethrin and pendimethalin the following procedure was applied.

The heterogeneous reactions of OH and O_3 with the pesticides, are illustrated by Eq. S9 and Eq. S10, respectively.

$$\text{Pesticides}_{\text{ads}} + \text{OH} \rightarrow \text{Products} \qquad [S9]$$

$$\text{Pesticides}_{\text{ads}} + \text{O}_3 \rightarrow \text{Products} \qquad [S10]$$

All degradation reactions of pesticides not initiated by OH radicals and O_3 can be defined as follows:

$$\text{Pesticides}_{\text{ads}} + \text{Y} \rightarrow \text{Products} \qquad [S11]$$

where Y signifies all the residual oxidant species.

The reaction rate of the pesticide under study in function of time is defined by Eq. S12.

$$-\frac{d[Pesticides_{ads}]}{dt} = k^{II}_{OH_{(part)}} \times [Pesticides_{ads}] \times [OH] + k^{II}_{O_3\,(part)} \times [Pesticides_{ads}] \times [O_3] + k^{II}_{Y_{(part)}} \times [Pesticides_{ads}] \times [Y] \qquad [S12]$$

where $k^{II}_{OH_{(part)}}$, $k^{II}_{O_3\,(part)}$ and $k^{II}_{Y_{(part)}}$ (cm^3 $molecule^{-1}$ s^{-1}) are the second order rate constants of the heterogeneous reactions with OH radicals, O_3 and all other species different than OH radicals and/or O_3.

The integration of Eq. S12 leads to:

$$ln\left(\frac{[Pesticides_{ads}]_t}{[Pesticides_{ads}]_0}\right) = -(k^{II}_{OH_{(part)}} \times [OH] + k^{II}_{O_3\,(part)} \times [O_3] + k^{II}_{Y_{(part)}} \times [Y]) \times t =$$

$$-k^{I}_{all_{(part)}} \times t \qquad [S13]$$

where $k^{I}_{all_{(part)}}$ (s^{-1}) is the pseudo first order constant of the heterogeneous reactions with OH radicals, O_3 and all other species different than OH radicals and/or O_3.

Then, the pseudo first order reaction rate constant $k^{I}_{OH_{(part)}}$ is expressed as follows:

$$k^{I}_{OH_{(part)}} = k^{II}_{OH_{(part)}} \times [OH] = k^{I}_{all_{(part)}} - k^{II}_{O_3\,(part)} \times [O_3] - k^{II}_{Y_{(part)}} \times [Y] \qquad [S14]$$

References

35. Pflieger, M., Monod, A. & Wortham, H. Heterogeneous oxidation of terbuthylazine by “dark” OH radicals under simulated atmospheric conditions in a flow tube. *Environ. Sci. Technol.* **47**, 6239–6246 (2013).

39. Lambe, A. T., Zhang, J., Sage, A. M. & Donahue, N. M. Controlled OH Radical Production via Ozone Alkene Reactions for Use in Aerosol Aging Studies. *Environ. Sci. Technol.* **41**, 2357-2363 (2007).

40. Le Person, A. *et al.* Trifluralin: Photolysis under sunlight conditions and reaction with HO radicals. *Chemosphere* **67**, 376-383 (2007).

41. Japar, S. M., Wu, C. H. & Niki, H. Rate constants for the reaction of ozone with olefins in the gas phase. *J. Phys. Chem.* **78**, 2318-2320 (1974).

42. Greene, C. R. & Atkinson, R. Rate constants for the Gas-Phase Reactions of O3 with a Series of Alkenes at 296 K. *Int. J. Chem. Kinet.* **24**, 803-811 (1992).

43. Witter, M., Berndt, T., Böge, O., Stratmann, F. & Heintzenberg, J. Gas-phase ozonolysis: Rate coefficients for a series of terpenes and rate coefficients and OH yields for 2-methyl 2-butene and 2,3-dimethyl-2-butene. *Int. J. Chem. Kinet.* **34**, 394-403 (2002).

44. Orzechowska, G. E. & Paulson, S. E. Production of OH radicals from the reactions of C4-C6 internal alkenes and styrenes with ozone in the gas phase. *Atmos. Environ.* **36**, 571-581 (2002).

45. Atkinson, R. & Aschmann, S. M. Rate constants for the reaction of OH radicals with a series of alkenes and dialkenes at 295 K. *Int. J. Chem. Kinet.* **16**, 1175-1186 (1984).

46. Atkinson, R. & Aschmann, S. M. Rate constants for the Gas-Phase Reactions of the OH Radicals with the Cresols and Dimethylphenols at 296 ± 2 K. *Int. J. Chem. Kinet.* **22**, 59-67 (1990).

47. Atkinson, R., Aschmann, S. M., Winer, A. M. & Pitts, J. N. Jr. Rate constants for the gas-phase reactions of OH radicals with a series of Alkanes and alkenes at 299 +/- 2 K. *Int. J. Chem. Kinet.* **14**, 507-516 (1982).

48. Atkinson, R., Aschmann, S. M. & Carter, W. P. L. Effects of ring strain on gas-phase rate constants. 2. OH Radical reactions with cycloalkenes. *Int. J. Chem. Kinet.* **15**, 1161-1177 (1983).

49. Atkinson, R., Arey, J., Aschmann, S. M., Corchnoy, S. B. & Shu, Y. Rate constants for the gas-phase reactions of cis-3-hexen-1-ol, cis-3-hexenylacetate, trans-2-hexenal, and linalool with OH and NO_3 radicals and O_3 at 296, and OH radical formation yields from the O_3 reactions. *Int. J. Chem. Kinet.* **27**, 941-955 (1995).

50. Ohta, T. & Ohyama, T. A set of rate constants for the reactions of OH radicals with aromatic hydrocarbons. *Bull. Chem. Soc. Jpn.* **58**, 3029-3030 (1985).

51. Hansen, D. A., Atkinson, R. & Pitts, J. N. Jr. Rate constants for the reaction of OH radicals with a series of aromatic hydrocarbons. *J. Phys. Chem.* **79**, 1763-1766 (1975).

52. Doyle, G. J., Lloyd, A. C., Darnall, K. R., Winer, A. M. & Pitts, J. N. Jr. Gas phase kinetic study of relative rates of reaction of selected aromatic compounds with hydroxyl radicals in an environmental chamber. *Environ. Sci. Technol.* **9**, 237-241 (1975).

53. Lloyd, A. C., Karen, R. D., Winer, A. M. & Pitts, J. N. Jr. Relative rate constants for reaction of the hydroxyl radical with a series of alkanes, alkenes and aromatic hydrocarbons. *J. Phys. Chem.* **80**, 789-794 (1976).

54. Perry, R. A., Atkinson, R. & Pitts, J. N. Jr. Kinetics and mechanism of the gas phase reaction of OH radicals with aromatic hydrocarbons over the temperature range 296- 473 K. *J. Phys. Chem.* **81**, 296-304 (1977).

55. Davis, D.D. Investigation of important hydroxyl radical reactions in the perturbed troposphere. U.S. Environmental Protection Agency, Washington, D.C., EPA/600/3-77/111 (1977).

56. Cox, R. A. & Derwent, R. Atmospheric photooxidation reactions. Rates, reactivity, and mechanism for reaction of organic compounds with hydroxyl radicals. *Environ. Sci. Technol.* **14**, 57-61 (1980).

57. Nicovich, J. M., Thompson, R. L. & Ravishankara, A. R. Kinetics of the reactions of the hydroxyl radical with xylenes. *J. Phys. Chem.* **85**, 2913-2916 (1981).

58. Atkinson, R. & Aschmann, S. M. Rate Constants for the Gas-Phase Reactions of the OH Radical with a Series of Aromatic Hydrocarbons at 296 K. *Int. J. Chem. Kinet.* **21**, 355-365 (1989).

59. Edney, E. O., Kleindienst, T. E. & Corse, E. W. Room-Temperature Rate Constants for the Reaction of OH with Selected Chlorinated and Oxygenated Hydrocarbons. *Int. J. Chem. Kinet.* **18**, 1355-1371 (1986).

60. Kramp, F. & Paulson, S. E. On the uncertainties in the rate coefficients for OH reactions with hydrocarbons, and the rate coefficients of the 1,3,5-trimethylbenzene and m-xylene reactions with OH radicals in the gas phase. *J. Phys. Chem. A* **102**, 2685-2690 (1998).

Table S1: The physico-chemical properties of the pesticides

Compounds	Cyprodinil	Deltamethrin	Difenoconazole	Fipronil	Oxadiazon	Pendimethalin	Permethrin	Tetraconazole
**CAS number**	121552-61-2	52918-63-5	119446-68-3	120068-37-3	19666-30-9	40487-42-1	52645-53-1	112281-77-3
**Chemical family**	Anilino-pyrimidine	Pyrethroïd	Triazole	Phenylpyrazole	Oxadiazole	Toluidine	Pyrethroïd	Triazole
**Nature**	Fungicide	Insecticide	Fungicide	Insecticide	Herbicide	Herbicide	Insecticide	Fungicide
**Molecular weight ($g\ mol^{-1}$)**	225.29	505.20	406.26	437.15	345.22	281.31	391.29	372.15
**Vapour pressure at 25°C (mmHg)** [a]	$3.68 \cdot 10^{-6}$	$1.50 \cdot 10^{-8}$	$2.50 \cdot 10^{-10}$	$2.78 \cdot 10^{-9}$	$1.12 \cdot 10^{-7}$ (22°C)	$3.00 \cdot 10^{-5}$	$2.18 \cdot 10^{-8}$	$1.35 \cdot 10^{-6}$ (20°C)
**Henry's constant at 25°C ($atm\ m^3\ mol^{-1}$)** [a]	$8.39 \cdot 10^{-8}$	$4.99 \cdot 10^{-6}$	$8.91 \cdot 10^{-12}$	$8.42 \cdot 10^{-10}$	$7.27 \cdot 10^{-8}$ (22°C)	$8.56 \cdot 10^{-7}$	$1.87 \cdot 10^{-6}$	$4.24 \cdot 10^{-9}$ (20°C)
**Molar volume ($cm^3\ mol^{-1}$) at 20°C and 760 Torr** [b]	186.1 ± 3.0	316.7 ± 3.0	287.1 ± 7.0	233.6 ± 7.0	262.4 ± 7.0	231.5 ± 3.0	302.5 ± 3.0	247.1 ± 7.0
**Characteristic ions (GC-(QqQ)-MS/MS)**	224.05<br>225.10<br>210.03	180.92<br>250.88<br>171.82	322.91<br>324.86	366.87<br>368.84<br>212.87	174.85<br>176.84<br>257.97	252.03<br>161.94	182.98<br>164.84	335.96<br>337.91

[a] SRC, PhysProp Database of Syracuse Research Corporation, estimate from VP/WSol or HENRYWIN: http://www.syrres.com/esc/physdemo.htm

[b] Scifinder, Calculated using Advanced Chemistry Development (ACD/Labs) Software V11.02 (© 1994-2014 ACD/Labs)
